# The Role of Hydrogen Sulfide in the Development and Progression of Lung Cancer

**DOI:** 10.3390/molecules27249005

**Published:** 2022-12-17

**Authors:** Yi-Lun Yang, Ka Zhang, Ze-Tao Zhou, Zhi-Liang Jiang, Yi Liu, Yan-Xia Zhang, Zhi-Hui Liu, Xin-Ying Ji, Dong-Dong Wu

**Affiliations:** 1 Henan International Joint Laboratory for Nuclear Protein Regulation, School of Basic Medical Sciences, Henan University, Kaifeng 475004, China; 2 School of Stomatology, Henan University, Kaifeng 475004, China; 3 School of Clinical Medicine, Henan University, Kaifeng 475004, China; 4 Department of General Practice, Henan Provincial People’s Hospital, People’s Hospital of Zhengzhou University, Zhengzhou 450003, China; 5 Faculty of Basic Medical Subjects, Shu-Qing Medical College of Zhengzhou, Zhengzhou 450064, China; 6 Kaifeng Key Laboratory for Infectious Diseases and Biosafety, School of Basic Medical Sciences, Henan University, Kaifeng 475004, China

**Keywords:** hydrogen sulfide, lung cancer, signaling pathway, molecular mechanism

## Abstract

Lung cancer is one of the 10 most common cancers in the world, which seriously affects the normal life and health of patients. According to the investigation report, the 3-year survival rate of patients with lung cancer is less than 20%. Heredity, the environment, and long-term smoking or secondhand smoke greatly promote the development and progress of the disease. The mechanisms of action of the occurrence and development of lung cancer have not been fully clarified. As a new type of gas signal molecule, hydrogen sulfide (H_2_S) has received great attention for its physiological and pathological roles in mammalian cells. It has been found that H_2_S is widely involved in the regulation of the respiratory system and digestive system, and plays an important role in the occurrence and development of lung cancer. H_2_S has the characteristics of dissolving in water and passing through the cell membrane, and is widely expressed in body tissues, which determines the possibility of its participation in the occurrence of lung cancer. Both endogenous and exogenous H_2_S may be involved in the inhibition of lung cancer cells by regulating mitochondrial energy metabolism, mitochondrial DNA integrity, and phosphoinositide 3-kinase/protein kinase B co-pathway hypoxia-inducible factor-1α (HIF-1α). This article reviews and discusses the molecular mechanism of H_2_S in the development of lung cancer, and provides novel insights for the prevention and targeted therapy of lung cancer.

## 1. Introduction

Lung cancer is a common malignant tumor that seriously threatens human life and health [1]. Its morbidity and mortality are at the top of the list in most countries [2]. As the early clinical symptoms of lung cancer are not obvious, most of the patients are diagnosed with a serious condition. According to the pathological classification, lung cancer can be divided into small-cell lung cancer (SCLC) and non-small-cell lung cancer (NSCLC). SCLC is the most malignant type of lung cancer, with an incidence of about 15–20%. NSCLC is mainly divided into squamous cell carcinoma, adenocarcinoma and large cell carcinoma, with an incidence about 80–85% [3,4,5]. At present, the main strategies for the treatment of lung cancer are surgery combined with radiotherapy and chemotherapy; however, the side effects are obvious. Therefore, it is urgent to develop novel methods for the treatment of lung cancer.

H_2_S is a new type of gas signal molecule after nitric oxide (NO) and carbon monoxide (CO) [6], and it is a colorless, flammable, and water-soluble gas with a rotten egg smell [7,8,9]. H_2_S is mainly produced by cystathionine β-synthase (CBS) and cystathionine γ-lyase (CSE) [10,11,12]. The third enzyme, 3-mercaptopyruvate sulfurtransferase (3-MST), can also generate endogenous H_2_S in the presence of a reductant using 3-mercaptopyruvate (3-MP) as substrate [13,14]. In addition, tobacco cigarette smoke, industrial gases, NaHS, GYY4137, AP39, etc., may produce exogenous H_2_S. H_2_S is reported to be involved in the development of many diseases [15] and it has both pro-apoptotic and anti-apoptotic effects in cultured cells [16,17]. H_2_S can participate in the occurrence and development of tumors through the mitogen-activated protein kinase-extracellular signal-regulated kinase (MAPK-ERK1/2) pathway, endoplasmic reticulum stress, and ion channels [18,19]. H_2_S has been shown to be involved in the occurrence and development of lung cancer [20]. In this article, the mechanism, therapeutic potential, and some unsolved problems of H_2_S and hydrogen sulfide donors in lung cancer are reviewed and discussed.

## 2. Synthesis and Metabolism of H_2_S in Lung Cancer

### 2.1. CSE

H_2_S is mainly produced by CBS and CSE [10,11,12]. The third enzyme, 3-MST, can also promote the production of endogenous H_2_S from 3-MP in the presence of reducing agents [13,14]. 3-MST exists in both mitochondria and cytoplasm, while CBS and CSE mainly exist in the cytoplasm [21]. Analysis of NSCLC biopsies and adjacent non-tumor tissues showed selectively high levels of endogenous H_2_S-producing enzymes, namely CBS, CSE, and 3-MST [22,23].

**Figure 1:**
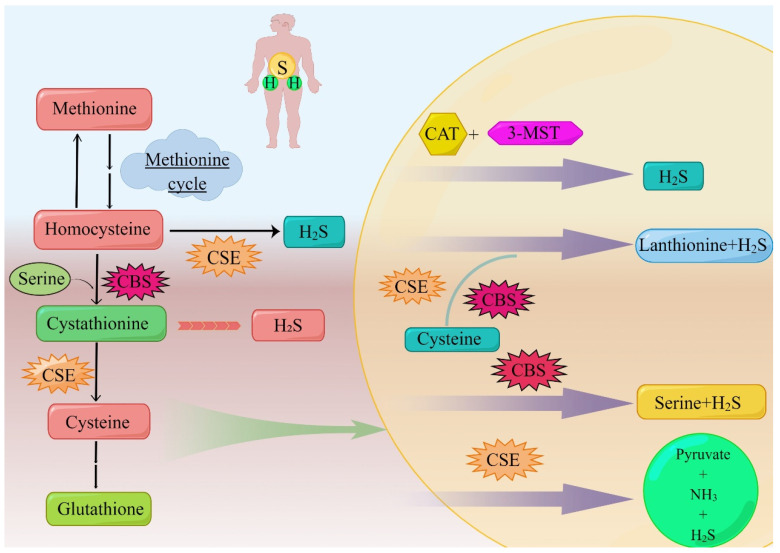
**The body produces H_2_S through the anti-sulfide pathway.** The sulfur transfer pathway plays an important role in redox regulation and cellular sulfur metabolism. The sulfur in this pathway is transferred from homocysteine to cysteine via mesocysteine, which is the only pathway for the endogenous production of cysteine in mammals. Methionine is converted to homocysteine in a reversible two-step process catalyzed by SAM and S-adenosine homocystease (not shown). Homocysteine is introduced into serine to produce cystathionine under the mediation of CBS, and this step can produce H_2_S. CSE can use homocysteine or cystathionine as substrate, the former to produce H_2_S, the latter to produce cysteine to continue the synthesis of H_2_S mediated by CSE, CBS, and 3-MST. Additionally, this part of the cysteine is involved in the synthesis of glutathione. 3-MST can cooperate with CAT to participate in the generation of H_2_S. CSE and CBS can mediate the generation of H_2_S with the addition of serine together, and they can also separately mediate the generation of H_2_S. Abbreviations; CBS: cystathionine β-synthase; CSE: cystathionine γ-lyase; 3-MST: 3-mercaptopyruvate sulfurtransferase; H_2_S: hydrogen sulfide; SAM: S-adenosylmethionine; GSH: glutathione; CAT: cysteine transaminase. (By Figdraw).

CSE is a homo-tetramer composed of pyridoxal 5′-phosphate (PLP)-bound 45 kDa subunits, and is the second enzyme to form H_2_S in the transsulfuration pathway. CSE mainly decomposes cysteine, a byproduct of CBS, into cysteine, α-ketobutyrate, and ammonia. Like CBS, CSE can also decompose cysteine and produce H_2_S (Figure 1). The PLP-CSE interaction is required for enzymic activity [23]. Hypoxia is a typical feature of solid tumors, including NSCLC [24]. CSE activity is a key driver of the transsulfuration pathway, cysteine catabolism, and H_2_S production. Tumor angiogenesis is induced by H_2_S mediated via hypoxia. H_2_S can increase the ability of endothelial cell invasion and duct formation [22]. H_2_S also plays a protective role in tumor-induced oxidative stress [25].

The typical role of CSE in the transsulfuration pathway is to cleave cysteine to form cysteine, ammonia, and α-ketobutyrate. Different from CBS, only homocysteine is used as the substrate of CSE in the formation of H_2_S [14]. Due to the low specificity of the CSE substrate, cystathionine, cysteine, and homocysteine can be regulated in the same binding capsule, where they compete with PLP to form Schiff base, while CBS has no binding site for PLP; therefore, H_2_S produced by CSE is more sensitive to homocysteine [12].

The protein and mRNA levels of CSE in tumor tissues are higher than in adjacent tissues. Compared with the corresponding levels in normal lung epithelial cell line BEAS-2B, NSCLC cell lines (A549 and 95D) showed the selective up-regulation of protein and mRNA expression of all three H_2_S-producing enzymes. It can be concluded that NSCLC cells selectively over-express CSE, thus inducing H_2_S production and promoting cell proliferation, migration, and invasion [22]. However, some studies have shown that compared with CBS, the clinical correlation between the expression of CSE in tumors and the prognosis of patients is not significant. There is no difference in clinical outcomes between high and low CSE expression in most cancers [26].

### 2.2. CBS

CBS is a homologous tetrameric enzyme with about 63 kDa subunits, which binds to two cofactors, PLP and heme [27]. The first and committed step in the transsulfuration pathway of the catalytic conversion of H_2_S by CBS is to use homocysteine instead of serine to form cystathionine and water. When the substrate is cysteine instead of serine, the products of the reaction are cysteine and H_2_S. CBS also catalyzes other reactions of cysteine to H_2_S [14]. Through the gene knockout experiment, it can be concluded that CSE is necessary for the synthesis of cysteine through sulfur transfer, and its gene destruction deprives CBS of one of the substrates needed to produce H_2_S. Thus, the disruption of CSE genes affects CSE- and CBS-dependent H_2_S synthesis and reduces H_2_S production. In contrast, disruption of the CBS gene results in the accumulation of homocysteine, a substrate for H_2_S production by CSE [10,14].

CBS exists mainly in the cytoplasm under normal physiological conditions [21]; however, CBS can be transferred to mitochondria in response to hypoxia or ischemia, a process that is partly the result of Lon protease regulating mitochondrial CBS stability [28]. Several studies have shown that hypoxia can be a condition that induces CBS translocation into the mitochondria. In fact, CBS can be detected in both the cytosolic and mitochondrial parts of HCT116 cells and A2780 cells [29,30]. The translocation of CBS to mitochondria may have important implications for the regulation of cancer cell bioenergy and survival [31]. It may be the result of threonine acylation that CBS enters the nucleus [32].

NSCLC cells selectively over-express CBS, which induces H_2_S production and promotes cell proliferation, migration, and invasion [22]. For example, in human non-small-cell lung adenocarcinoma, Western blot analysis of tumor tissue compared with normal adjacent lung tissue (n = 20) showed that CBS protein is significantly increased about five-fold in tumor homogenate; furthermore, these tissues also produce approximately two times as much H_2_S as their surrounding normal tissues [33]. Increased levels of CBS protein or mRNA have also been reported in two different collections of lung cancer clinical specimens [8]. Hypoxia, glucose deprivation, and hydrogen peroxide treatment have been applied to various HCC and breast cancer cells to generate repaired cells, which are partially resistant to subsequent injury and exhibit the up-regulation of CBS; therefore, in this experimental model, oxidative stress seems to be the most important factor leading to the up-regulation of CBS [34,35]. CBS mRNA and protein levels are slightly up-regulated in chemically hypoxic A549 lung cancer cells treated with cobalt chloride [22]. Lung adenocarcinoma cell lines A549, H522, H1944, Calu-6 cells, A549, and 95D cells are used to examine the functional role of CBS. The results show that the expression of CBS in cancer cell lines is significantly higher than that in the untransformed lung epithelial cell line Beas2B control [8,22,33]. Studies have shown that CBS mRNA is induced in response to the transcription factor Nrf2 [36], and CBS protein levels are regulated by both transcriptional and post-transcriptional processes, including ubiquitination [31].

### 2.3. 3-MST

3-MST is a 33 kDa zinc-dependent enzyme and H_2_S-producing enzyme. As early as 1959, Hylin and Wood confirmed that 3-MST could produce polysulfides [37]. Kamoun thought that 3-MST had the ability to produce H_2_S as early as 2004. 3-MST is a PLP-independent enzyme that catalyzes L-cysteine to form H_2_S. In addition, 3-MST can bind to cysteine aminotransferase in the presence of α-ketoglutarate [38].

3-MST is structurally expressed in all kinds of somatic cells, as well as various cancer cells. Wrobel’s team has performed related earlier studies on the expression of 3-MST in cancer cells: in human tumor cell lines (U373 astrocytoma cell line; SH-SY5Y neuroblastoma cell line; and two melanoma cells lines, A375 and WM35), they found a large amount of 3-MST expression and enzymatic activity. The expression and activity of 3-MST in these cell lines are significantly higher than those of CSE, so it can be concluded that 3-MST is a more important source of H_2_S [39,40]. Western blotting indicates [33] that 3-MST expression is either considerably greater or marginally higher in human lung cancer tumors compared with adjacent non-cancer tissues. However, the expression of 3-MST in human papillary thyroid carcinoma tumors is not different from that in surrounding non-cancer tissues [41], and the expression level of 3-MST in renal cell carcinoma tumors is highly variable [42]. At present, a variety of lung adenocarcinoma cell lines (A549 H522~H1944) have been proven to have 3-MST expression/catalytic activity [33,43].

One of the characteristics of lung cancer is angiogenesis, and this process can supply the oxygen and nutrients required for the growth of lung cancer cells; furthermore, it also creates the potential for tumor spread. It is reported that H_2_S has the ability to promote new blood vessels [44,45]. Related studies have shown that 3-MST-derived H_2_S produced by endothelial cells plays a certain role in vasodilation, endothelial cell proliferation, migration, and angiogenesis, especially under hypoxic conditions [13,46,47,48]. Some studies have shown that silent 3-MST can reduce the proliferation rate of A549 cells (human lung adenocarcinoma cell line) and decrease the repair rate of mitochondrial DNA [37]. To summarize, 3-MST participates in the generation and development of lung cancer and plays an important role. Furthermore, we compare CSE, CBS, and 3-MST in Table 1.

### 2.4. Catabolism of H_2_S in Lung Cancer

CSE and CBS are primarily expressed in human airway smooth muscle cells (SMCs), pulmonary blood vessels and endothelial cells, and the airway SMCs of mouse lung. Some studies have shown a higher expression of CSE in the airway and peripheral lung tissue of rat pulmonary blood vessels [53]. The primary steps in H_2_S catabolism include exhalation, lung ventilation, methylation modification, and oxidation. Large quantities of H_2_S cause the mitochondria to generate thiosulfate, which is converted to sulfate by rhodanese. Thiol S-methyltransferase, on the other hand, catalyzes the methylation of H_2_S in the cytoplasm, where it is transformed into methanethiol and dimethyl sulfide [23]. Methanethiol is converted into dimethyl sulfide by S-methyltransferase. In conclusion, H_2_S may be quickly expelled from the body as a gas in the respiratory system and can be eliminated in the urine as sulfate and thiosulfate.

## 3. Mechanism of Endogenous H_2_S in Lung Cancer

### 3.1. CSE

CSE can be transferred from cytoplasm to mitochondria under cellular stress, such as an increase in intracellular Ca^2+^ levels [29,30,49]. Under hypoxia, changes in cellular ion channels will lead to an increase in intracellular Ca^2+^ levels [54], which promotes the transfer of CSE to mitochondria. This process is mediated by adventitia transferase 20 (Tom20) [49]. CSE translocation to mitochondria contributes to the reduction of mitochondrial cysteine through dehydration [55]. The metabolites of cysteine, such as H_2_S, act as antioxidants [25]. In SMC, CSE is the only enzyme that produces H_2_S in vascular smooth muscle cells. Increasing ATP synthesis is a method of mitochondrial CSE transport and H_2_S generation to improve hypoxic tolerance. However, considering that most cancer cells use the energy of aerobic glycolysis, CSE mitochondrial translocation in cancer cells may not be necessary [49]. In addition, CSE can be modified by small ubiquitin-like modified protein (SUMO). SUMO is usually considered as a nuclear location signal [32,56].

Endogenous H_2_S produced by CSE can also act as a bioenergy stimulant [31]. CSE activity is a key driver of the transsulfuration pathway, cysteine catabolism, and H_2_S production [23]. Silencing H_2_S synthase, especially CSE, can inhibit the epithelial–mesenchymal transition (EMT) process of NSCLC cells [22].

### 3.2. CBS

Studies have shown that in human non-small-cell lung adenocarcinoma, tumor cells express more CBS than adjacent normal lung tissue cells [33].

H_2_S produced by CBS is used to (I) support tumor growth and proliferation by maintaining the energy supply of colon cancer cells and (Ⅱ) provide blood and nutrients to tumors by promoting angiogenesis and vasodilation. According to the current research results, CBS-derived H_2_S is identified as the target of tumor growth factor and anticancer drugs [29].

CBS catalyzes several reactions, typical of which is the β-displacement reaction of L-serine with L-homocysteine to form L-cysteine and water without H_2_S production. CBS can catalyze a variety of H_2_S formation reactions, including the condensation of L-cysteine and L-homocysteine to form L-cysteine sulfide and H_2_S. The condensation of two L-cysteine molecules to form L-blue-thionine and H_2_S-L-homocysteine. The β-γ replacement reaction of L-homocysteine with L-cysteine to form L-cysteine sulfide and H_2_S. L-cysteine persulfide is formed from L-cysteine. Furthermore, L-cysteine persulfide releases H_2_S or partially transfers sulfane sulfur to the receptor protein in the presence of a reducing agent [31]. It has been found that the control or inhibition of CBS can reduce the intracellular content of the essential antioxidant glutathione (GSH + GSSG) and lead to the apoptotic cascade [57]. Meanwhile, p53 is increased and the expression of NF-KB-RelA/p65 subunits is decreased. CBS inhibition can also increase mitochondrial ROS production, decrease the NAD/NADH ratio, reduce ATP synthesis, and increase the ADP/ATP ratio. Mitochondrial oxygen consumption can be reduced by CBS [30]. Loss of CBS blocks the density and crimp of CD31-positive blood vessels between tumor tissues, indicating decreased tumor angiogenesis [29].

Overall, the cells overexpressing CBS show higher metabolic, proliferative, invasive, dedifferentiated dry state, chemo-resistant, and immune cell resistance phenotypes. In tumor cells with high CBS expression, silencing or inhibition of CBS can obtain “mirror” biological responses, such as the inhibition of cell proliferation, invasion, and cell bioenergetics [31].

### 3.3. 3-MST

3-MST is one of the endogenous H_2_S synthase and is mainly expressed in mitochondria. There may be two molecular mechanisms of 3-MST-mediated H_2_S formation: (i) The nucleophilic cysteine (Cys247) of sulfur is transferred from 3-MP to its active site to form a stable persulfide. Then, the persulfide is attacked by sulfur-containing compounds to form a new persulfide molecule, which is reduced by thioredoxin (Trx) to produce H_2_S. (ii) When the substrate is a dithiol compound, some sulfur atoms may be reduced to H_2_S by the self-reduction process [58,59,60].

3-MST exists in the equilibrium form of monomer dimmer and its C-terminal has catalytic activity. Cys247 is the catalytic site, while Cys154 and Cys263 are located on the surface of the enzyme. Cys247 has redox activity and can be oxidized to sulfonyl cysteine, which in turn is reduced to the active form by Trx. Cys247 is used as a redox sensor switch in the subunit. H_2_S is produced by 3-MST in the presence of Trx or dihydrolipoic acid (DHLA). 3-MST reacts with 3-MP to form H_2_S through the reaction of persulfide intermediates. Trx or DHLA receives a sulfur atom from the persulfide, which is attacked by another mercaptan and releases H_2_S [58,59,60]. Cys154 and Cys263 are involved in the formation of intermolecular disulfide and affect the activity of the enzyme. 3-MST contains a key catalytic site, cys247, which is sensitive to redox [58]. Therefore, oxidative stress has been shown to inhibit the catalytic activity of 3-MST, thus inhibiting the production of H_2_S mediated by 3-MST [50,51,52]. The mechanism of 3-MST in vasodilation and angiogenesis is related to the regulation of the 3-MST system in endothelial cell bioenergetics and metabonomics [48]. Existing data show that 3-MST can play an effective role in the treatment of lung cancer; however, its mechanism has not been clearly explored and more research is needed.

## 4. Mechanism of Exogenous H_2_S in Lung Cancer

### 4.1. NaHS

Although smoking is the main cause of lung cancer, World Health Organization data show that about 75% of lung cancer patients are smokers, which means that about 25% of lung cancer patients are non-smokers. The data suggest that there are other risks that can lead to lung cancer. At present, epidemiology has shown that nickel compounds can increase the mortality of lung cancer [61].

Nickel is a common environmental factor and can be found in soil and water resources [62]. Nickel compounds are common environmental and occupational carcinogens. Recent studies have shown that nickel chloride plays a certain role in the migration of lung cancer cells [63]. In the process of the invasion and migration of tumor cells, epithelial cells will produce EMT in different degrees. EMT refers to the process of epithelial cells losing apical polarity and intercellular adhesion into cells with an interstitial phenotype, which plays a key role in the development of lung cancer [64]. Nicl2 is reported by many studies as inducing the EMT process of A549 cells [65]. Recently, several studies have shown that exogenous H_2_S is feasible in promoting cancer radiotherapy and anticancer therapy [66,67]. A total of 100 μM NaHS pretreatment can alleviate the morphological changes of A549 cells induced by NiCl2 and also alleviate the effect of NiCl2 treatment on the protein levels of E-cadherin and vimentin. Therefore, NaHS can inhibit the migration of A549 cells by preventing the EMT of A549 cells [65].

GF-β1 plays a regulatory role in a variety of biological processes [67], while TGF-β1 has important anticancer effects in early tumorigenesis and mediates EMT [68]. Some studies have shown that NiCl_2_ can up-regulate the levels of TGF-β1, p-Smad2, and p-Smad3 in a time-dependent manner, while exogenous NaHS reduces the up-regulation of NiCl_2_-dependent TGF-β1, p-Smad2, and p-Smad3 [65]. These results show that NaHS can inhibit the migration ability of A549 cells induced by NiCl_2_ via the TGF-β1/Smad2/Smad3 signal pathway. In addition, the migration and invasion of A549/DDP cells can be inhibited by enhancing their sensitivity to cisplatin [66]. Therefore, NaHS is expected to be used as a therapeutic strategy for the treatment of cisplatin-resistant NSCLC.

### 4.2. GYY4137

GYY4137 is not only a water-soluble compound, but also one of the donors of H_2_S. H_2_S can be released by hydrolysis [69,70]. In the experiment of exploring the effects of NaHS and GYY4137 on cell growth and viability, Zheng Wei Lee et al. treated MCF-7, MV4-11, and HL-60 cancer cells with NaHS, GYY4137, and ZYJ1122. It was found that GYY4137 significantly decreased the proliferation of these three cancer cells compared with NaHS and ZYJ1122. In addition, they used 400 mM and 800 mM of GYY4137 and ZYJ1122 to treat other cancer cell lines (HeLa, HCT-116, USOS, and HepG2 cells). The results showed that GYY4137 has a more profound effect on cell survival at the same concentration, with a mortality rate of 30–70% for all cancer cell lines treated with GYY4137, and has the greatest impact on the death courses caused by HepG2, HL-60, MV4-11, MCF-7, and U2OS cells [71].

Some studies have proposed that GYY4137 slowly decomposes to produce H_2_S, which inhibits tumor growth by blocking the cell cycle and promoting cell apoptosis [71]. Although there is no related research on the role of GYY4137 in the treatment of lung cancer, according to the data we have collected, GYY4137 may provide new ideas for the treatment of lung cancer in the future.

### 4.3. AP39

The repair of mitochondrial DNA (mtDNA) can be regulated by H_2_S. It has been discovered that mtDNA encodes a group of proteins necessary for preserving oxidative phosphorylation. In many malignancies, somatic mtDNA mutations contribute to the selective advantage of carcinogenesis [72,73,74].

According to recent reports, the degree of mtDNA damage can influence the loss of cancer cells’ capacity to proliferate to some amount [75]. In a study, Bartosz Szczesny’s team demonstrated that inhibition of tumor H_2_S production leads to time-dependent accumulation of mtDNA damage in A549 cells, and inhibition of H_2_S biosynthesis makes A549 cells more susceptible to mtDNA oxidative damage [33]. To counteract oxidative mtDNA degradation, A549 cells might receive the mitochondrial-targeted H_2_S donor (AP39) [33]. As a result, we are aware that H_2_S is crucial in avoiding oxidative mtDNA damage and can improve mtDNA repair. Another study revealed that AP39 performs a stimulatory function in mtDNA repair and safeguards mtDNA integrity in endothelial cells under oxidative stress [76]. According to the data, AP39 has the function of stimulating mitochondrial electron transport and cellular bioenergy at low concentrations (30–100 nM), while they are inhibited at high concentrations (300 nM) [52,76]. This is in line with H_2_S having an effective response that has a bell-shaped distribution (many actions are helpful and benign at low doses but harmful to cells at high concentrations) [77,78].

**Figure 2:**
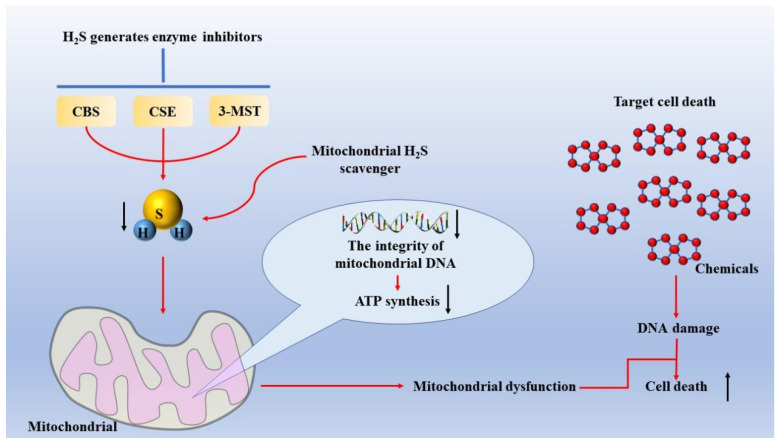
**Mechanism of inducing mitochondrial dysfunction in lung adenocarcinoma cells.** H_2_S-generating enzyme inhibitors, such as AOAA, which is commonly used in CBS and CSE, or mitochondrial H_2_S scavengers, are used to reduce intracellular H_2_S levels, thereby inducing mitochondrial dysfunction and improving the sensitivity of lung adenocarcinoma cells to chemotherapy drugs. Abbreviations: AOAA: aminooxyacetic acid; CBS: cystathionine β-synthase; CSE: cystathionine γ-lyase.

In conclusion, certain H_2_S-generating enzyme inhibitors or specific H_2_S clearance agents paired with chemotherapeutic medicines that decrease mitochondrial DNA repair capacity and oxidative phosphorylation can cause mitochondrial malfunction. As a result, chemotherapy medications particularly affected lung adenocarcinoma cancer cells (Figure 2), increasing the effectiveness of the therapy [33]. More research is required since there are few data on H_2_S and mtDNA, and the molecular mechanism of mtDNA integrity preservation in cancer cells has not been thoroughly examined.

## 5. Mechanism of H_2_S Donor in Natural Products in Lung Cancer

### 5.1. DATS

Diallyl trisulfide (DATS) is one of the garlic-derived compounds, accounting for 45% of garlic oil, and has anti-tumor, anti-inflammatory, immunomodulatory, and chemical-preventive effects [79]. In cancer chemoprevention, DATS can reduce the induction rate of carcinogens and inhibit the proliferation and activity of various cancer cells. Studies have shown that its mechanism may be related to cell cycle arrest, induction of apoptosis, and the regulation of carcinogenic signal transduction pathway [80]. At present, it has been reported that DATS has a significant anticancer effect on lung cancer [81].

Cisplatin (DDP) is a common chemotherapeutic drug in cancer treatment, which plays a significant role in tumor treatment; however, its side effects also limit its extensive application in tumor treatment, such as acute kidney injury (AKI) induced by DDP administration [82]. In the process of tumorigenesis, unlimited cell proliferation is one of the markers of tumorigenesis; therefore, the induction of cell cycle block can be used as a target for the treatment of cancer [83]. Members of the Bcl-2 family play an important role in the regulation of apoptosis [84]. The activation of caspase and the activities of PI3K/Akt, MAPK/ERK, MAPK/JNK, and MAPK/p38 pathways play an important role in the proliferation and metastasis of cancer cells [85]. The related research report of Xiaoyan Jiang’s team [81] showed: (i) Through the verification of in vivo and in vitro experiments, the results showed that DATS combined with DDP can enhance the anti-tumor ability by inducing apoptosis; (ii) after DATS treatment of NCI-H460 cells, the proportion of apoptotic cells increased in a dose-dependent manner, so DATS has good anti-proliferative activity for NCI-H460 lung cancer cells [81]. The mechanism is to stabilize the cell cycle in the G1 phase. It is related to the increase in the intracellular G1 phase and the decrease in the intracellular G2max M phase [86]; (iii) ATS+DDP treatment has fewer side effects, such as improving the oxidative damage induced by DDP treatment. After DATS treatment, the expression of E-cadherin increased and the expression of MMP-9 decreased, which contributed to the inhibition of the EMT process; (iv) the apoptosis of NCI-H460 cells induced by DATS mainly depends on the activation of caspase and regulates the inhibition of Bcl-2 family proteins; (v) the combination of DDP and DATS can increase the activity of DATS and increase the apoptosis of tumor cells.

**Figure 3:**
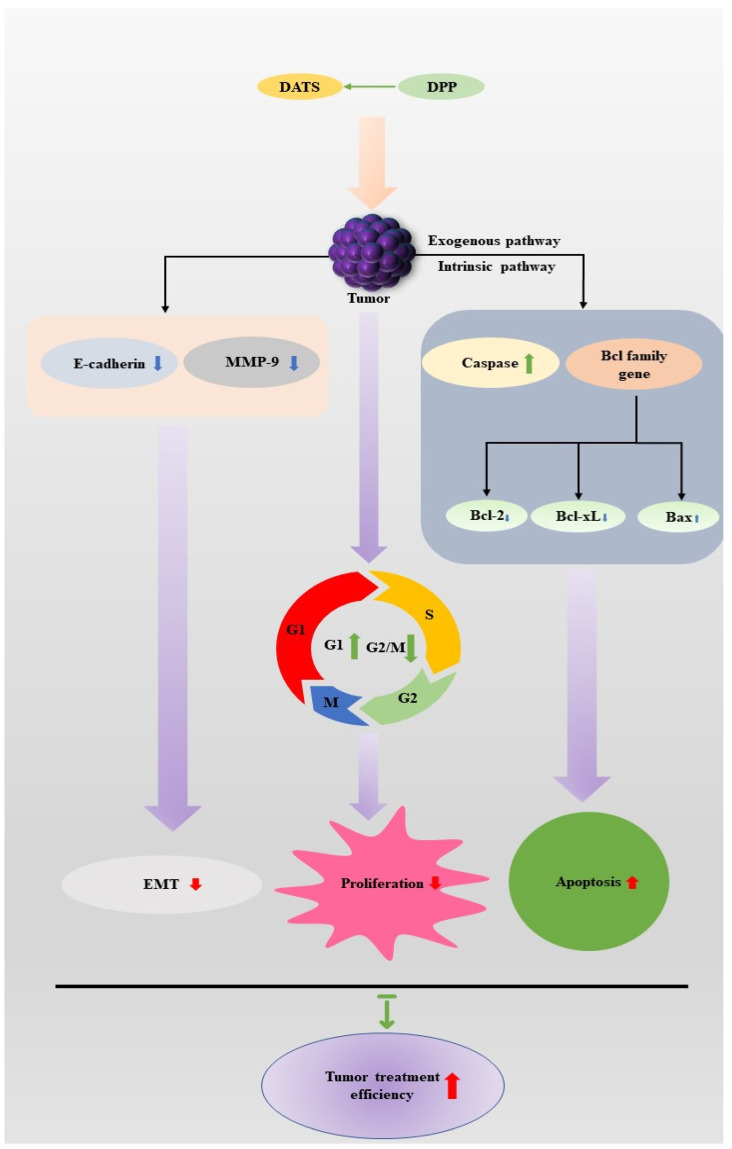
**Therapeutic mechanism of DATS combined with DDP in lung cancer cells.** When DATS is combined with DDP, DDP can enhance the activity of DATS. After DATS treatment of lung cancer tumors, the expression level of E-cadherin increased and the expression level of MMP-9 decreased, which inhibited the EMT process in tissues. At the same time, DATS activates caspases through endogenous and exogenous pathways and inhibits Bcl-family proteins, among which the expression level of pro-apoptotic factor Bax increases and the expression level of pro-growth factors Bcl-xl and Bcl-2 decreases, thus promoting the apoptosis of tumor cells. In addition, DATS increased the level of G1 phase and decreased the level of G2/M phase in tumor cells to inhibit the proliferation of tumor cells. Through the above process, the efficiency of lung cancer treatment can be improved. Abbreviations: DATS: diallyl trisulfide; DDP: cisplatin.

In summary, DATS plays an important role in the treatment of lung cancer, and the combination of DATS and DDP may provide a new idea for the clinical treatment of lung cancer (Figure 3).

### 5.2. DADS

A large number of studies have confirmed that garlic and its allyl sulfides have anti-tumor effects, and diallyl disulfide (DADS) is a fat-soluble active ingredient, accounting for about 60% of garlic oil. Studies have shown that DADS can inhibit plaque formation and reduce the risk of hypertension and coronary artery disease [87]. In recent years, a number of studies have shown that DADS can inhibit tumorigenesis induced by a variety of carcinogens and inhibit the formation of various tumor cells. Additionally, DADS can induce apoptosis of human leukemia, colon cancer, prostate cancer, and breast cancer cells [88]. In addition, some studies have shown that DADS can induce apoptosis in NSCLC H1299 cells [89].

**Figure 4:**
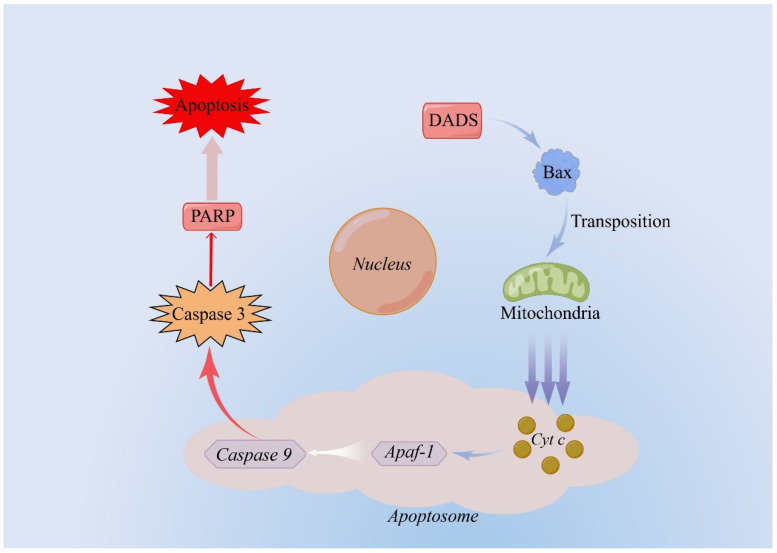
**The molecular mechanism of apoptosis induced by DADS.** DADS stimulates the translocation of pro-apoptotic protein Bax from cytoplasm to mitochondrial membrane, which increases the permeability of mitochondrial membrane and promotes the release of cytochrome C from mitochondria. Apoptotic enzyme activator binds with cytochrome C to form a polymer, and Caspase-9 is recruited and processed to form an apoptotic complex. Finally, the downstream apoptosis factor Caspase-3 is activated, which leads to apoptosis. Abbreviations: DADS: diallyl disulfide. (By Figdraw).

Recently, the results of quantitative reverse transcriptase polymerase chain reaction and Western blot analysis showed that DADS induced caspase-dependent apoptosis of human cancer cells through Bax (Figure 4). Therefore, it can be speculated that the mitochondrial pathway may be used as a target for DADS in chemoprevention or chemotherapy [90]. Hui Cao’s team explored the effect of DADS on human NSCLC H1299 cells [89]. Their results showed that: (i) DADS can induce apoptosis of the H1299 cells; (ii) DADS can block the G2 phase of H1299 cell cycle; (iii) DADS-induced apoptosis depends on the classical mitochondrial pathway; (iv) H1299 cells were treated with DADS at concentrations of 0, 20, 50, and 100 μmol/L, respectively. After 10 min, it was found that the increase in phosphorylated-p42hammer 44 is concentration-dependent. The results showed that the activation of phosphorylated p42/44MAPKs plays an important role in the mechanism of apoptosis induced by DADS [89].

At present, the study of H_2_S in the treatment of lung cancer has become a hot topic. In view of the low toxicity of DADS and its inhibitory effect on a variety of tumor cells, an in-depth study of the mechanism of DADS in lung cancer will be of great significance for the clinical treatment of DADS in lung cancer.

### 5.3. DAS

Diallyl sulfide (DAS) is not only an effective component of garlic but also a lipophilic sulfide, which can be oxidized to diallyl sulfoxide and diallyl sulfone mediated by cytochrome P450 enzymes [91]. It selectively inhibits and induces some P450 enzymes [91,92,93,94], which play a certain role in tumor treatment and chemical prevention. In addition, DAS can inhibit DNA binding and AFB1-, vinyl carbamate-, and NDMA-related cancer-induced mutations [95,96].

NNK is a potent tobacco carcinogen and is one of the important causes of oral cancer in tobacco chewers and lung cancer in smokers [97]. Some studies have shown that DAS can inhibit the oxidative metabolism of NNK in the lungs of mice in a dose-dependent manner [98]. In research, a team used the lung cancer model of A/J mice to show that DAS inhibits NNK metabolism. When DAS 200 mg/kg was given 3 days and 2 h before a single NNK treatment, the tumor incidence was significantly reduced by 60% and the tumor proliferation rate was significantly reduced by 90% [98]. In addition, it has been reported that DAS can inhibit the occurrence of lung adenoma induced by bp [99].

At present, it is known that DAS or its metabolites may inhibit the activity of metabolic enzymes in NNK partly by reducing metabolic activation; however, the therapeutic mechanism of DAS in lung cancer remains to be studied.

## 6. Therapeutic Strategies for Targeting H_2_S in Lung Cancer

In the past few decades, anticancer drugs have mostly been cytotoxic compounds. Although these drugs have good efficacy in the treatment of cancer, some of them cannot distinguish normal cells from cancer cells, so they also have side effects on the human body (such as DDP-administration-induced AKI). With the development of molecular biology and genomics, molecular targeted therapy for cancer has attracted more and more attention [100]. The advantages of molecular targeted therapy over traditional anticancer drugs are [101]: (i) targeted killing of tumor cells while basically not damaging normal cells and (ii) less toxicity than traditional anticancer drugs. Among them, H_2_S as a targeted drug has also attracted much attention in the treatment of lung cancer.

Overexpression and activation of IDO1 in tumor and antigen-presenting cells can produce toxic tryptophan metabolites and play an important role in tumor-induced immune system tolerance and inhibition, which has become a new important therapeutic target [102,103,104]. Dang Yang ‘s team found that H_2_S can down-regulate IDO1 expression by blocking NF-κB and STAT3 pathways and inhibit IDO1 activity through H_2_S/NO cross-talk, which has an immunotherapeutic effect on H22 hepatocellular carcinoma (HCC) tumor-bearing mice [105]. In addition, they found that H_2_S can also inhibit the activity of IDO1 in CSE^−/−^ mice and MCF-7 and SGC-7901 cells [105]. It has been reported that nosh-aspirin (a mixture that releases NO and H_2_S) significantly inhibits lung adenocarcinoma cells [106]. Thus, H_2_S has the potential to inhibit IDO1 and is expected to be used in the treatment of lung cancer. In addition, it has been reported that the introduction of a H_2_S-release structure to valproic acid (VPA) can more potently inhibit the growth and metastasis of lung cancer cells, increasing the sensitivity of lung cancer cells to chemotherapeutic drugs [107].

At present, the specific mechanism of H_2_S-targeted therapy in lung cancer has not been studied clearly, but from the existing research we can know that H_2_S-targeted therapy for lung cancer has great potential. In addition, H_2_S-based treatment technology can provide new ideas for future lung cancer treatment.

## 7. Conclusions and Future Prospect

H_2_S is a new type of gas signaling molecule that has attracted much attention in recent years and is widely involved in the regulation process of the respiratory system and digestive system. H_2_S plays a key role in the regulation of cell activity (such as oxidative stress, apoptosis, cell differentiation, and inflammation). It may play a significant role in the occurrence and development of lung cancer, in which the increase of H_2_S level is closely related to angiogenesis and EMT in lung cancer tissue. Inhibition of H_2_S-producing enzymes by specific scavengers or reduction of intracellular H_2_S levels can lead to mitochondrial dysfunction and make lung adenocarcinoma cells sensitive to chemotherapeutic drugs. However, the molecular mechanism of how H_2_S regulates the integrity of mitochondrial DNA and how these processes play an important role in the tumorigenic potential of cancer cells is not clear [27,33]. However, it has been reported that both endogenous and exogenous H_2_S may regulate mitochondrial energy metabolism and mitochondrial DNA integrity and participate in the inhibition of lung cancer cells through a variety of signaling pathways. The above different results may be explained by the clock effect of H_2_S. For a long time, the bell-shaped effect of H_2_S has attracted much attention. H_2_S has a certain concentration range to promote tumor cell proliferation, which can be achieved by reducing the production of endogenous H_2_S or the application of exogenous H_2_S for therapeutic purposes. Among them, there are many reports on the use of exogenous H_2_S to treat cancer (or disease). It has been reported that when exogenous H_2_S inhibits the concentration of tumor cells, normal tissue will be damaged. Additionally, some studies have found that cancer cells can be selectively killed when exposed to a relatively small amount of H_2_S for a relatively long time; therefore, the optimal time and concentration of H_2_S in the treatment of lung cancer remain to be studied. In addition, there are many reports that hydrogen sulfide inhibitors and the addition of H_2_S release structures play a significant role in the treatment of lung cancer and are expected to be developed into clinical anticancer drugs. Endogenous H_2_S is mainly synthesized by CBS, CSE, and 3-MST. The elevation of enzyme expression in lung cancer cells and the synthesis of H_2_S have been widely reported; however, there are still few studies on the use of the endogenous H_2_S synthesis pathway and H_2_S pathway in the treatment of lung cancer. At present, the problem of how H_2_S (3-mst-derived or other) interacts with various components of the cell microenvironment has not been solved. Therefore, further research is needed to establish a literature basis for the possibility of H_2_S as a diagnostic tool or therapeutic target.

In addition, numerous studies have reported that some H_2_S donors and some components of garlic can effectively inhibit the proliferation of lung cancer cells and can be combined with other anticancer drugs to reduce side effects or increase drug activity (for example, sodium hydrosulfide increases the sensitivity of cisplatin against cisplatin in lung cancer cells, and DATS and DDP increase the therapeutic effect). However, because of the complexity, the mechanism and specific function of its action have not been studied clearly.

Taken together, the role of H_2_S in tumor prevention, detection, and treatment has great potential; however, there are still many challenges, including the complexity of the free diffusion of H_2_S into the cell membrane, remaining to be studied. In order to find new therapeutic targets, a large number of experimental studies are needed to confirm and deepen the mechanism and role of the H_2_S donor combined with DDP in the treatment of lung cancer. It is expected to provide new ideas for the treatment of lung cancer. In addition, we believe that a clear study of the mechanism of H_2_S in the development of lung cancer and its interaction with tumor cell microenvironment can provide new therapeutic methods and means for the treatment of lung cancer.

## Figures and Tables

**Table 1:** Abbreviations: CSE: cystathionine γ-lyase; EMT: epithelial–mesenchymal transition; H_2_S: hydrogen sulfide; CBS: cystathionine β-synthase; NSCLC: non-small-cell lung cancer; 3-MST: 3-mercaptopyruvate sulfurtransferase.

Table	Location	Transfer	Expression and Activity	The Effect after Inhibition
CSE	Cytoplasm [21]	CSE can be transferred from the cytoplasm to the mitochondria in response to cellular stress, such as increased intracellular Ca^2+^ levels [29,30,49].	The clinical correlation between CSE expression in tumor and patient prognosis was not significant. In most cancers, there was no difference in clinical outcomes between high and low CSE expression [26].	Silencing H_2_S synthase, especially CSE, inhibits the EMT process in NSCLC cells [22].
CBS	Cytoplasm [21]	Hypoxia may be a condition that leads to the translocation of CBS into mitochondria, which is of great significance for the regulation of biological energy and survival in cancer cells [28,31].	Oxidative stress seems to be the most important factor leading to the upregulation of CBS. Cells with overexpression of CBS show higher metabolism, proliferation, aggressiveness, dedifferentiated dry state, chemotherapy resistance, and immune cell resistance [8,22,33].	CBs-derived H_2_S has been identified as a target for tumor growth factors and anticancer drugs, and loss of CBS blocks the density and curl of CD31-positive blood vessels between tumor tissues, indicating reduced tumor angiogenesis [29].
3-MST	Mitochondria and cytoplasm [21]	Lack of coverage.	Cancer cell lines with 3-MST expression/catalytic activity include various lung adenocarcinoma cell lines. 3-MST is involved in the occurrence and development of lung cancer and plays an important role. 3-MST can play an effective role in the treatment of lung cancer; however, its mechanism is not clear and more research is needed [48].	H_2_S synthesis decreased after 3-MST inhibition [50,51,52].

## Data Availability

Not applicable.
